# The potential benefit of sodium glucose cotransporter (SGLT)-2 inhibitors in liver cirrhosis: A mini review

**DOI:** 10.1177/17562848261485144

**Published:** 2026-09-29

**Authors:** Michelle Spaan, Abraham S. Meijnikman, Daniel H. van Raalte, Joost P. H. Drenth

**Affiliations:** 1 Department of Gastroenterology and Hepatology, Amsterdam University Medical Center, Amsterdam, The Netherlands; 2 Department of Internal Medicine, Amsterdam University Medical Center, Amsterdam, The Netherlands

**Keywords:** SGLT2-inhibitors, cirrhosis, renal function, acute kidney injury, hepatorenal syndrome, liver decompensation

## Abstract

Liver cirrhosis represents a growing global health burden and prognosis is determined by the severity of portal hypertension and the development of complications. Among these, kidney dysfunction stands out as one of the strongest predictors of mortality. Sodium–glucose cotransporter-2 (SGLT2) inhibitors have demonstrated robust kidney and cardiovascular benefits across diverse populations, and early evidence suggests potential applicability in cirrhosis. This mini review synthesizes current knowledge on the natural history of kidney function decline in cirrhosis, modifiable risk factors, and the emerging rationale for SGLT2 inhibitor therapy in this high-risk population.

**Key messages**

- AKI is frequently observed in patients with cirrhosis and is among the strongest prognostic indicators for disease progression and survival.
- SGLT2i promote glucosuria and transient natriuresis and have proven kidney protective effects in patients with type 2 diabetes, heart failure and CKD.
- Real-world data increasingly suggest that SGLT2i may be associated with improved clinical outcomes in patients with cirrhosis
- However, it remains uncertain whether patients with cirrhosis benefit from SGLT2i and if kidney or hepatic outcomes may be affected by treatment.
- Addressing these uncertainties, well-designed randomized controlled trials are essential.

## 1. Introduction

Liver cirrhosis affects approximately 120 million adults worldwide PMID^1^ and has increased by nearly 60% over the past three decades.^2^ It is the third leading cause of death among individuals aged 45–64 years, reflecting a substantial mortality risk.^3^ The prognosis of liver cirrhosis is primarily determined by the presence of hepatocellular carcinoma and portal hypertension-related complications such as ascites, variceal hemorrhage and hepatic encephalopathy. Renal complications like acute kidney injury (AKI) and hepatorenal syndrome (HRS) are common and are associated with high morbidity and mortality.^4^ Kidney function consistently emerges as a key determinant of survival. This is reflected in the Model for End-Stage Liver Disease (MELD), where the level of serum creatinine heavily influences liver transplant prioritization. Despite this central role in prognosis of chronic liver disease, kidney function in cirrhosis is often conceptualized as a static variable: an outcome of liver disease rather than a therapeutic target. With this review we call for a shift in perspective: kidney dysfunction in cirrhosis is dynamic, modifiable, and amenable to intervention.

## 2. Pathophysiology of kidney function in cirrhosis

In the general population, renal function (eGFR) naturally declines with age by approximately 0.4–1.1 mL/min/1.73 m^2^ per year, averaging around ∼1 mL/min/1.73 m^2^ annually in healthy adults with faster decline seen at older ages and with comorbidities, particularly in those with increased urinary albumin excretion.^5^ Cirrhosis is an important risk factor for eGFR decline.^6^ Here, splanchnic vasodilation reduces effective arterial blood volume and activates neurohormonal vasoconstrictor systems, including renin–angiotensin–aldosterone system (RAAS). In the non-cirrhotic population, angiotensin II causes afferent and mainly efferent renal vasoconstriction but due to a lack of vasodilators (e.g. prostaglandins) in cirrhosis, afferent renal vasoconstriction predominates, causing a progressive decline in eGFR and hepatorenal dysfunction.^4^ In the general population, after activation of RAAS, angiotensin II also causes proximal sodium and water reabsorption (e.g. via NHE3 – MAP17- PDZK1) thereby maintaining renal blood flow. In non-cirrhotic individuals, reduced sodium delivery to the macula densa triggers tubuloglomerular feedback (via NKCC2), resulting in dilation of the afferent arteriole and suppression of renin release, thereby attenuating RAAS activation. In contrast, in decompensated cirrhosis, persistent renal vasoconstriction and impaired renal perfusion maintain a reduced glomerular filtration rate (GFR), leading to sustained RAAS activation and continued sodium and water retention.^7^

Growing evidence suggests that kidney function in cirrhosis does not typically follow a linear, gradual decline but rather follows a complex trajectory with repeated episodes of AKI, and progression to CKD.^4,8,9^ Since AKI develops in 20–40% of hospitalized patients with cirrhosis,^10,11^ it is not surprising that patients with cirrhosis show a steeper and less predictable decline in kidney function compared to non-cirrhotic controls.^12,13^ Despite the clear relevance of preserving kidney function, longitudinal cohort studies evaluating kidney function decline in patients with cirrhosis are scarce. Large-scale datasets offer snapshots of kidney function at discrete time points but fail to capture the true natural history or identify critical inflection points at which interventions may be most effective. As a result, prediction of kidney decline remains imprecise, highlighting a major unmet need.

## 3. Kidney function is a prognostic marker in cirrhosis

Kidney function is among the strongest prognostic indicators in cirrhosis. Kidney impairment is associated with high morbidity and mortality, prolonged hospitalizations and higher healthcare costs.^14,15^ In a cohort of 3.6 million hospital admissions for cirrhosis, 22% had AKI and despite advances in supportive care, outcomes remain poor.^14^ Recovery of kidney function occurs in only 59% of hospitalized patients with cirrhosis complicated by AKI.^10^ Even after apparent recovery of eGFR, persistent subclinical structural injury, nephron loss, and reduced functional renal reserve frequently remain, increasing susceptibility to progressive CKD.^10,15^ AKI in cirrhosis holds strong prognostic value: cirrhotic admissions complicated by AKI have a ∼3.75-fold higher odds of in-hospital death (adjusted OR 3.75, 95%CI 3.71–3.79) compared to non-AKI admissions.^14^ Also in liver transplant recipients the etiology and severity of AKI at the time of the surgical procedure are strong determinants of ultimate renal outcomes.^16,17^ Because AKI severity (stage ≥1B) is an independent predictor of subsequent CKD in cirrhosis, interventions that attenuate AKI severity are likely to confer long-term renal benefit.^15^

## 4. Modifiable risk factors for kidney function decline in cirrhosis

Kidney dysfunction in cirrhosis is often multifactorial and can be exacerbated by several modifiable risk factors. In this paragraph we highlight a number of these risk factors. While kidney function decline may occur without an obvious precipitating event, it is frequently triggered by conditions that reduce effective intravascular blood volume. Key contributors in cirrhosis are gastrointestinal bleeding, large-volume paracentesis (LVP), volume depletion induced by diuretics such as spironolactone or furosemide, and poor oral intake in relation to vomiting or diarrhea. Close monitoring of kidney function and early adjustment of therapy is essential to prevent AKI and mitigate progression to CKD in this population. Diabetes mellitus type 2 (T2DM) increases the risk of kidney dysfunction by 2- to 3-fold and is highly prevalent among patients with MASLD-related cirrhosis.^18^ Poor glycemic control accelerates kidney microvascular injury, leading to increased inflammation and oxidative stress, and persistent hyperglycemia drives glomerular hyperfiltration, further impacting the kidney. Therefore, strict metabolic control is critical, not only to manage blood glucose levels but more so to preserve kidney function.^19^

Hemodynamic dysregulation also contributes to impaired kidney perfusion. Here the therapeutic dilemma comes at full display: overtreatment with non-selective beta-blockers may lower mean arterial pressure (MAP) to below 65 mmHg, compromising kidney perfusion, while undertreatment may allow persistent portal hypertension which reduces effective arterial blood volume and threatens to worsen kidney hypoperfusion. Balancing these conflicting effects remains a significant clinical challenge.

Additional modifiable risk factors include the exposure to nephrotoxic agents such as radiocontrast dyes, aminoglycosides and vancomycin, as well as infections, particularly from spontaneous bacterial peritonitis or urinary tract infections. While managing of these risk factors is vital, such interventions alone are often insufficient to prevent progressive kidney dysfunction in patients with cirrhosis. As our understanding of hepatorenal interaction evolves, new therapeutic strategies come into play.

**Figure 1.**
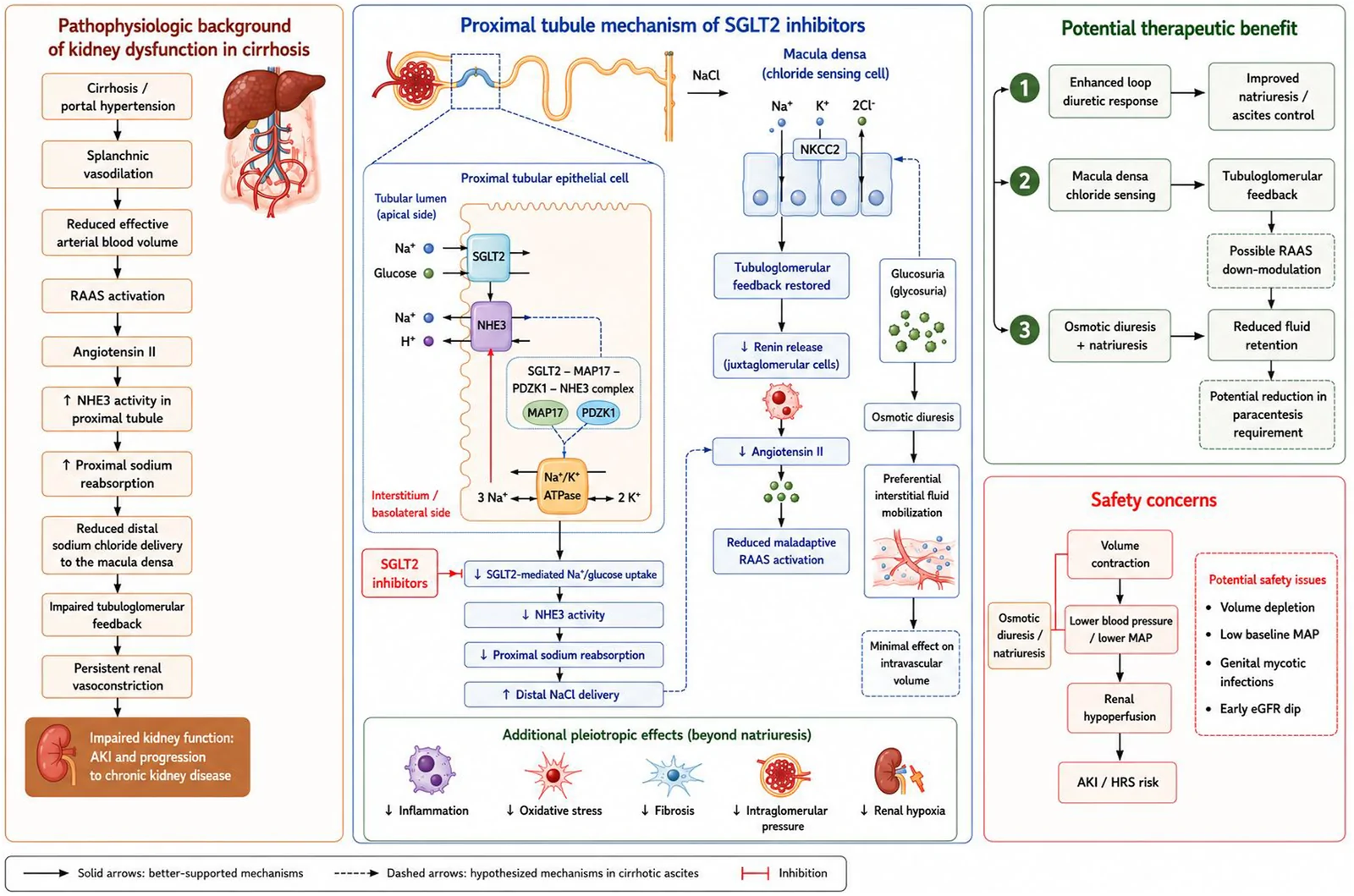
Potential benefit of SGLT2-inhibitors in cirrhosis. Cirrhosis-induced neurohumoral activation increases proximal tubular sodium reabsorption, reduces distal sodium chloride delivery, and impairs tubuloglomerular feedback, promoting renal vasoconstriction and kidney dysfunction. SGLT2 inhibition decreases proximal sodium and glucose reabsorption, reduces NHE3 activity, restores tubuloglomerular feedback through enhanced macula densa chloride sensing, and attenuates maladaptive RAAS activation. Together with osmotic diuresis and additional anti-inflammatory, antifibrotic, and anti-hypoxic effects, these mechanisms may improve natriuresis, ascites control and kidney outcomes. Potential adverse effects include volume depletion, hypotension, and genital mycotic infections. Solid arrows indicate established mechanisms; dashed arrows indicate proposed mechanisms in cirrhosis.

## 5. SGLT2 inhibitors: Mechanistic rationale in cirrhosis

Sodium–glucose cotransporter-2 inhibitors (SGLT2i), originally designed for the treatment of T2DM, inhibit SGLT2 receptor which is located in the proximal tubule where they reduce proximal tubular glucose reabsorption and induce glucosuria. A recent review proposed that the renoprotective effects of SGLT2i are mediated through functional coupling between the sodium–hydrogen exchanger 3 (NHE3) and SGLT2 in the proximal tubule. Inhibition of SGLT2 reduces proximal tubular sodium reabsorption, increasing sodium and chloride delivery to the macula densa, thereby restoring tubuloglomerular feedback and modulating RAAS activity.^20,21^ Notably, in the DAPASALT studies, dapagliflozin did not increase urinary sodium excretion in patients with chronic kidney disease (CKD), suggesting that its effects are not mediated through sustained natriuresis or a conventional diuretic mechanism.^22^ Instead, enhanced sodium and chloride delivery to the macula densa is thought to restore tubuloglomerular feedback, attenuate maladaptive RAAS activation, and preserve renal function.^22^ Since this the main mechanisms of kidney dysfunction in patients with cirrhosis, these treatments have high potential to result in kidney protection in this patient population. Other mechanism of action of SGLT2i are a reduction in inflammation, oxidative stress, fibrosis, glomerular pressure and protection from kidney hypoxia.^23,24^

Inhibition of SGLT2 in the proximal tubule reduces glucose and sodium reabsorption, leading to glucosuria and osmotic diuresis. Unlike conventional diuretics, SGLT2i appear to preferentially mobilize interstitial rather than intravascular fluid, thereby promoting decongestion while preserving effective arterial blood volume.^25^ This mechanism may be particularly relevant in patients with cirrhosis, in whom renal dysfunction is frequently aggravated by diuretic resistance and circulatory dysfunction. Consequently, SGLT2i may improve volume management while reducing the requirement for conventional diuretics, potentially lowering the risk of diuretic-related kidney dysfunction. In addition, their use is associated with weight loss and improved insulin sensitivity which may benefit particularly patients with MASLD-driven cirrhosis. The potential mechanisms of SGLT2i in patients with cirrhosis are summarized in Figure 1.

These mechanisms contribute to their clinically significant kidney protective effects across a broad range of patient population including T2DM,^26^ heart failure irrespective of ejection fraction,^27^ and CKD^28^ and is currently also being investigated in patients undergoing cardiac surgery as a short-term perioperative treatment to prevent AKI.^29^ Three landmark randomized controlled trials have established the kidney protective efficacy of SGLT2i demonstrating a 30% relative risk reduction in end-stage kidney disease (ESKD), or renal death.^30–32^ Importantly, two meta-analysis of 12 and 18 large RCT’s also found a 25% reduction in AKI among those with CKD with similar reductions in patients with and without T2DM.^28,33^ Which is especially relevant since AKI is the leading contributor of CKD development in cirrhosis.^4^

## 6. Current evidence for SGLT2 inhibitors in liver disease

Large real-world datasets provide emerging evidence suggesting potential clinical benefits of SGLT2i in patients with cirrhosis. A cohort study using data from 120 healthcare organizations within the TriNetX platform in the U.S.A included patients with cirrhosis who received SGLT2i as well as furosemide and spironolactone. These patients were matched with a control group of patients who were receiving furosemide and spironolactone alone, according to age, demographics, and comorbidities using 1:1 propensity matching. Each patient was followed up for 3 years. Among 10.660 propensity-matched patients, complementary SGLT2i therapy was associated with a significantly lower incidence of serious liver events defined as incidence of ascites, variceal development, hyponatremia, or all-cause mortality. (HR 0.68, 95%CI 0.66-0.71, p<0.001). Secondary outcomes included, among others, hepatorenal syndrome (HR, 0.47 [95% CI, 0.40-0.56]), paracentesis (HR, 0.54 [95% CI, 0.50-0.60]), and all-cause hospitalizations (HR, 0.67 [95% CI, 0.63-0.71]), all of which were associated with a reduced risk among those in the SGLT-2i group. In addition, the risk of hepatorenal syndrome seem to be reduced compared to controls (HR 0.47, 95%CI 0.40-0.56).^34^

Consistent findings were reported in an independent large propensity-matched cohort study using the same TriNetX platform, including all patients with compensated cirrhosis and T2DM. Patients with estimated GFR ≤ 30 mL/min/1.73 m^2^ were excluded from analyses since this is a contraindication for metformin use. Propensity matched patients receiving dual therapy with metformin and an SGLT2i (n=1.403) had decreased risk for all-cause mortality (HR 0.57, 95%CI 0.41–0.81) compared to patients receiving metformin alone. Secondary outcomes included hepatic decompensation, defined as a composite of ascites, hepatic encephalopathy, and esophageal or gastric variceal bleeding. The risk of hepatic decompensation was lower among patients with type 2 diabetes and cirrhosis receiving dual therapy compared with those receiving monotherapy (HR 0.63, 95% CI 0.43–0.93).^35^ There were no data on development of AKI or CKD in both groups.

Further support comes from a nationwide population-based cohort study using data from a health insurance database. This study included 399.126 patients with diabetic MASLD (DM-MASLD), in which (15.7%) used a SGLT2i. After propensity score matching for age, sex, antidiabetic use, obesity, the presence of diabetic complications, hyperlipidemia and hypertension, SGLT2i use was associated with a reduced risk of developing CKD (HR 0.66, 95%CI 0.62-0.70, p<0.001), end-stage kidney disease (HR 0.61, 95%CI 0.48-0.77, p<0.001), but also incident cirrhosis (HR 0.80, 95%CI 0.76-0.84, p<0.001) and hepatic decompensation (HR 0.79, 95%CI 0.76-0.84, p<0.001).^36^

Since SGLT2 is a 1:1 cotransporter of glucose and sodium, the administration of SGLT2i to patients with T2DM promotes glucose excretion to the tubule causing osmotic water excretion. In line, various case reports have reported favorably on the effect of SGLT2i on the management of (refractory) ascites in patients with cirrhosis.^37–39^ The reduction of ascites in patients using SGLT2i was also observed in two randomized placebo-controlled pilot studies. One study from India included 40 patients with cirrhosis and recurrent ascites. Control of ascites was significantly better after 6 months of treatment with dapagliflozin compared to the standard of care group (*p* < 0.04).^40^ In another small trial from Egypt including 42 patients with cirrhosis and refractory ascites (median MELD-Na score in SGLT2i group 18), patients showed a reduction in the need for LVP after 3 months of treatment with empagliflozin versus the standard of care group (100% need for LVP in the standard of care vs. 42.9% in the empagliflozin group).^41^

Collectively, these findings suggest that SGLT2i use in cirrhosis may be associated with a lower incidence of CKD, end-stage kidney disease, and hepatorenal syndrome, as well as fewer hepatic decompensation events and improved ascites control. Nonetheless, most evidence is observational and susceptible to confounding by indication, and the available RCT’s are small and short-term with some signals of increased AKI incidence reported, warranting caution in this hemodynamically vulnerable population. All studies used in this review a summarized in Supplemental table 1.

## 7. Safety of SGLT2 inhibitors in patients with cirrhosis

While the data on the safety of SGLT2i in patients with liver disease remain limited, the majority of the literature is consistent with the absence of direct hepatotoxicity in Child-Pugh A and B patients.^42^ Empagliflozin in particular, has been tested in patients with advanced fibrosis and compensated cirrhosis, and demonstrated to have a favorable safety profile.^35,43^ Pharmacokinetic studies indicate that while empagliflozin exposure increases with worsening hepatic impairment, the increase is less than twofold, and single-dose administration is well tolerated across Child-Pugh A–B classifications.^44–46^ Both dapagliflozin and empagliflozin have increased systemic exposure in Child-Pugh C cirrhosis and long-term safety has not been established.

One of the main safety concern is the reduction of mean arterial blood pressure by SGLT2i seen in multiple large RCT and meta-analysis.^47^ A meta-analysis (2019, 7 RCT’s, n=2.381) with 24 hour out-patient blood pressure measurements showed average reduction of 3.62 mmHg systolic and 1.70 mmHg diastolic in patients treated with SGLT2i compared to low dose hydrochlorothiazide.^48^

Some caution is advisable when prescribing SGLT2i in patients with advanced cirrhosis, as their natriuretic and osmotic diuretic effects may contribute to volume depletion and hypotension, potentially precipitating AKI.^45,46^ In the previously described pilot study from India, AKI occurred more frequently in patients receiving SGLT2i than in those receiving standard of care (50% vs 15% after 6 months, p=0.04).^40^ Although nearly all AKI episodes in the Indian study were precipitated by sepsis (12/13) or variceal bleeding (1/13),^40^ this finding represents a potential safety signal that warrants further investigation. In the other small pilot study from Egypt, AKI was more prevalent in the standard of care group.^41^ Interpretation of these results is limited by the small sample sizes and relatively short treatment duration. Large randomized trials in patients with CKD, T2DM, and heart failure have consistently shown that SGLT2i cause an early, transient decline in estimated glomerular filtration rate (eGFR), reflecting a hemodynamic reduction in intraglomerular pressure, followed by sustained long-term kidney protective effects.^49^ In clinical trials of autosomal dominant polycystic kidney disease (ADPKD), tolvaptan, a selective vasopressin V2 receptor antagonist, restored tubuloglomerular feedback and induced an early decline in estimated glomerular filtration rate (eGFR), consistent with a reduction in glomerular hyperfiltration. Importantly, this initial eGFR decrease was not associated with AKI or a sustained loss of kidney function. On the contrary, long-term treatment with tolvaptan slowed the rate of eGFR decline by approximately 1.3 mL/min/1.73 m^2^ per year and has consequently received international regulatory approval for the treatment of ADPKD.^50^

Importantly, since infections and sepsis is a major threat to patients with cirrhosis, large cardiovascular and renal outcome trials have not demonstrated an increased risk of serious infections with SGLT2i, including urinary tract or respiratory infections, although genital mycotic infections are more common due to glucosuria.^51,52^ In the pilot study from India however, more infections were reported in the dapagliflozine group compared to standard of care group (55% vs 20%, p=0.04).^40^ Future randomized controlled trials should incorporate rigorous safety monitoring, predefined stopping criteria, and standardized protocols for temporary drug interruption and re-initiation to better define the safety profile of SGLT2i in patients with cirrhosis.

## 8. Conclusion

Kidney dysfunction is central to prognosis in cirrhosis yet remains under-recognized as a modifiable therapeutic target. Multiple reversible factors accelerate kidney function decline, but even with optimal management, patients remain at high risk. SGLT2i represent a biologically plausible approach to kidney protection in cirrhosis, with early studies suggesting potential benefits and no major safety concerns identified to date. However, key questions remain regarding the target population with cirrhosis that may benefit best from SGLT2i. It is unclear whether potential beneficial effects are dependent on the presence of clinically significant portal hypertension, or whether benefits are confined to those with T2DM or may also extend to non-diabetic cirrhosis. Addressing these uncertainties will require well-designed randomized controlled trials to determine the impact of SGLT2i on clinically relevant outcomes including the development of CKD, prevention of AKI and liver-related decompensation. As the burden of cirrhosis grows, therapeutics that jointly address metabolic dysfunction, inflammation, hemodynamics, and renal protection may reshape the management of cirrhosis in the coming decade.

## Supplemental material

Supplemental material - The potential benefit of sodium glucose cotransporter (SGLT)-2 inhibitors in liver cirrhosis: A mini review Supplemental material for The potential benefit of sodium glucose cotransporter (SGLT)-2 inhibitors in liver cirrhosis: A mini review by Michelle Spaan, Abraham Meijnikman, Daniel H. van Raalte and Joost P. H. Drenth in Therapeutic Advances in Gastroenterology.

## Appendix

### Abbreviations

AKI: acute kidney injury
CKD: chronic kidney disease
eGFR: estimated glomerular filtration rate
HRS: hepatorenal syndrome
MAP: mean arterial pressure
NHE3: sodium–hydrogen exchanger 3
RAAS: renin–angiotensin–aldosterone system
SGLT2: sodium–glucose cotransporter 2
NKCC2: sodium–potassium–2 chloride cotransporter.
