# Supplementary material for: The potential benefit of sodium glucose cotransporter (SGLT)-2 inhibitors in liver cirrhosis: A mini review
Source: Ther Adv Gastroenterol. 2026 Sep 29;19:17562848261485144. doi: 10.1177/17562848261485144 (PMC13769902; doi:10.1177/17562848261485144)
Supplement: Supplementary file 1 [file sj-pdf-1-tag-10.1177_17562848261485144.pdf]

**Supplementary Figure 1. SGLT2 Inhibitors in Cirrhosis — Summary of Key Clinical Studies used in this review**

| Study | Design | N | Cirrhosis stage | Etiology | Diabetes | Child-Pugh / MELD | Ascites phenotype | Agent vs comparator | Primary outcome / key results | Safety signals & limitations |
|---|---|---|---|---|---|---|---|---|---|---|
| Abu-Hammour et al.<br>JAMA Netw Open 2025 | Retrospective cohort<br>TriNetX, PS-matched<br>3-yr follow-up | 10,660 matched (5,330 vs 5,330) | Any stage | Any etiology | ~80% T2DM<br>in both arms | Not reported | Present, on furosemide+ spironolactone | SGLT2i class (agents unspecified)<br>+ diuretics vs diuretics alone | Primary composite (ascites/ varices/hyponatremia/ mortality) HR 0.68 (0.66–0.71). HRS HR 0.47; SBP HR 0.55; paracentesis HR 0.54; variceal bleed HR 0.79 | Retrospective, ICD-10 misclassification, generalizability limited to diabetic/diuretics-treated |
| Huynh et al.<br>BMC Gastroenterol 2023 | Retrospective cohort<br>TriNetX, PS-matched<br>5-yr follow-up | 1,403 matched (11411 SGLT2i + metformin<br>22,515 metformin alone) | Compensated only | Mainly MASH (40%) | 100% | MELD-Na 6-7 in both arms. | Not applicable | SGLT2i (mix)<br>+ metformin vs metformin alone | 5-yr mortality HR 0.57 (0.41–0.81); decompensation HR 0.63 (0.43–0.93); HCC HR 0.43 (0.21–0.88). | AKI/CKD not assessed. No ascites-specific outcome. |
| Singh et al.<br>Dig Dis Sci | RCT, double-blind,<br>placebo-controlled,<br>1:1, 6-mo follow-up | 40<br>(20 vs 20) | Decompensated; Child-Pugh B/C | Mainly ALD (50%) | 5-10% | Median MELD-Na 22 | Recurrent ascites (NOT formally “refractory” per ICA criteria) | Dapagliflozin 10mg/d<br>+ SoC vs SoC alone | Ascites control at 6mo better w/ dapa (complete 15%+partial 55% vs complete 0%+partial 35%, p=0.01–.04); urine Na↑ (p<0.001). NO diff.<br>in CTP/MELD or survival (65% vs 72.2%, p=0.75) | AKI significantly HIGHER w/ dapa (50%<br>vs 15%, p=0.04); infections higher (55%<br>vs 20%, p=0.04). Pilot, no power calc, single-center, short follow-up |
| Bakosh et al.<br>Egypt Liver J<br>2024 | RCT, 3-mo follow-up | 42<br>(21 vs 21) | Decompensated; refractory ascites (explicit) | Mainly HCV and SLD | 40-50% | Median MELD-Na 14-18 | Refractory ascites | Empagliflozin 10mg<br>+ SoC vs SoC alone | No need for LVP: 42.9% (empa) vs 100% (SoC), p<0.001; ~24% total ascites resolution by study end | SoC-alone arm had a higher (non-significant) AKI rate |
| Colletta et al. Front Endocrinol, 2025 | Retrospective cohort study, 48 months | 54 (27 vs 27) | CP-B cirrhosis | 100% MASLD | 100% | Median MELD-Na 12-13. CP-8-9 | ~70 ascites | SGLT2i vs insulin | GFR increased in the SGLT2i group (+13.5 ± 1.3) and declined in the insulin group (−4.2 ± 1.4; p < 0.01). A greater proportion of SGLT2i patients transitioned from CKD stage 3a to 2 (p = 0.04). Improvement of MELD in SGLT2 group | Compared to insulin, small sample size |
| Shen et al. Br J Clin Pharmacol, 2024 | Open label single arm, 4 weeks | 10 (5 compensated, 5 decompensated) | Compensated and decompensated | 60% Alcohol, 30% HCV | Not reported | 100% CP-A in compensated group, 80% CP-B and 20% CP-C in decompensated group. MELD-NA 8 vs 14 | 0% in compensated vs 20% in decompensated | Empagliflozine single arm | Primary aim: safety, tolerability, PK and PD of empagliflozin . Adverse events in 8/10 (80%); serious AEs in 3/10 (30%), one attributed to empagliflozin (hypotension) | Small n=10, no control arm, short exposure (4 wk) |
| Kasichayanula *et al.*, Eur J Int Med, 2011 | Open-label single arm, 3 months | 14 | Decompensated cirrhosis with newly diagnosed refractory ascites | alcoholic/viral/immune: 8/4/2 | 100% T2DM | CP B/C: 10/4 | Diuratic retractable 10 patients, diuretica resistant in 4 patients | single arm | Ascites was sufficiently controlled in all patients. Dosages of diuretics were significantly lower (p<0.001 for furosemide) than that prior to enrollment. GFR increased significantly (p<0.001) | Small n=14, no control arm, short exposure, one patient died due to pneumonia |
| Macha *et al.*, Diabetes Obes Metab, 2014 | Open-label, single-dose pharmacokinetic study | 36 cirrhosis + 12 matched healthy controls | 8 CP-A + 8 CP-B + 8 CP-C | Not reported | 25-29% T2DM | 8 CP-A + 8 CP-B + 8 CP-C. MELD not reported | Not reported | Single 50 mg dose of empagliflozin | Primary aim: effect of hepatic impairment on empagliflozin PK, safety, tolerability. Empagliflozin rapidly absorbed with biphasic plasma decline; exposure (Cmax/AUC) not meaningfully altered by hepatic impairment severity in a way requiring dose adjustment | Single-dose design (no steady-state or ascites/efficacy data), supratherapeutic dose, small subgroup sizes (n=8/arm); foundational PK safety data only, |
